# Free access to medicines among older adults in primary care: a cross-sectional study

**DOI:** 10.1590/1516-3180.2019.0541.R1.19022020

**Published:** 2020-06-22

**Authors:** Isabela Vaz Leite Pinto, Marina Guimarães Lima, Laís Lessa Neiva Pantuzza, Maria das Graças Braga Ceccato, Micheline Rosa Silveira, Adriano Max Moreira Reis

**Affiliations:** I MSc. Pharmacist, Municipal Health Department, Municipal Government of Belo Horizonte, Belo Horizonte (MG), Brazil.; II PhD. Associate Professor and Pharmacist, School of Pharmacy, Universidade Federal de Minas Gerais (UFMG), Belo Horizonte (MG), Brazil.; III MSc. Doctoral Student and Pharmacist, School of Pharmacy, Universidade Federal de Minas Gerais (UFMG), Belo Horizonte (MG), Brazil.; IV PhD. Associate Professor and Pharmacist, School of Pharmacy, Universidade Federal de Minas Gerais (UFMG), Belo Horizonte (MG), Brazil.; V PhD. Associate Professor and Pharmacist, School of Pharmacy, Universidade Federal de Minas Gerais (UFMG), Belo Horizonte (MG), Brazil.; VI PhD. Associate Professor and Pharmacist, School of Pharmacy, Universidade Federal de Minas Gerais (UFMG), Belo Horizonte (MG), Brazil.

**Keywords:** Aged, Primary health care, Public health, Pharmaceutical preparations, Pharmaceutical services, Elderly, Medicines, Access to medicine

## Abstract

**BACKGROUND:**

Access to medicines is an important indicator of healthcare system quality and capacity to resolve problems. The healthcare system needs to ensure free access to medicines for elderly people, in order to provide greater effectiveness of disease control, thus reducing morbidity and mortality, and improving health and quality of life.

**OBJECTIVES:**

To analyze the frequency of free access to medication among older adults within primary care and determine the factors associated with free access.

**DESIGN AND SETTING:**

Cross-sectional study at two primary care units.

**METHODS:**

Free access was defined as provision of all medicines through pharmacies within the Brazilian National Health System and through the Brazilian program for free medicines in private pharmacies. We investigated the sociodemographic, clinical, functional and pharmacotherapeutic characteristics of older adults. Multivariate logistic regression was performed to identify factors associated with free access to medicines.

**RESULTS:**

This study included 227 older adults, among whom 91 (40.1%) had free full access to prescription drugs. A direct association with age ≤ 70 years and indirect associations with polypharmacy and multimorbidity (P < 0.05) were found.

**CONCLUSIONS:**

Age ≤ 70 years increases the likelihood of having free full access to medicines, and older adults with multimorbidity and polypharmacy use have a lower likelihood of access. Identification of factors associated with free access to medicines among elderly people provides elements to guide the Brazilian National Health System in implementing access improvement actions.

## INTRODUCTION

The contribution of medicines to better quality of life, recovery of health and increased survival is appreciable.^1^ Thus, resolute public healthcare actions to curb morbidity and mortality are strongly influenced by rational access to and use of drugs.^1^^,^^2^^,^^3^^,^^4^

Given the increasing burden of chronic noncommunicable diseases around the world, access to medicines encompassing availability and affordability is a significant public health challenge for both developed and developing countries. The global action plan for noncommunicable disease prevention and control coordinated by the World Health Organization recommends that actions to expand access to medicines should be developed.^5^^,^^6^

In Brazil, through the National Drug Policy, measures have been implemented to ensure and expand the population’s access to medicines since 1998. In 2004, actions were scaled up when the National Pharmaceutical Services Policy Guidelines were defined.^4^^,^^7^ The following stand out among the different strategies for improving access to medicines that are underway in this country: structuring of pharmaceutical services; improvement and innovation of the legal framework for accessing medicines within the Brazilian National Health System; improved organization of financing for public pharmaceutical services; and higher levels of federal resources for procurement of medicines.^4^

Brazil has been experiencing aging of the population as a result of the epidemiological and demographic transition that is taking place in this country. This situation greatly affects the planning of social and healthcare policies, given the increased prevalence of older adults with multiple noncommunicable diseases requiring complex multidrug therapy.^1^^,^^8^^,^^9^ The National Survey on Drug Access, Use and Promotion of Rational Use in Brazil found that 75.7% of older adults used two or more chronic drugs.^1^ Given this scenario, healthcare systems need to ensure free access to medicines for elderly people, in order to provide greater effectiveness of disease control, thus reducing morbidity and mortality, and improving health and quality of life.^3^^,^^4^^,^^5^^,^^6^

Access to medicines is an important indicator of healthcare system quality and capacity to resolve problems and is considered by the United Nations to be an appropriate means for measuring progress towards achievement of healthcare rights.^7^^,^^10^^,^^12^^,^^13^

Primary healthcare is provided through an extensive network of services and can solve most of a population’s health problems. Consequently, ensuring free access to medicines for older adults at this level of care should be a priority.^7^^,^^9^

## OBJECTIVE

The aims of this study were to analyze the frequency of free access to medication among elderly people attended at two primary healthcare centers and to determine the factors associated with free access.

## METHODS

### Study design and participants

This was a cross-sectional study conducted at two primary care units (PCUs) located in the city of Belo Horizonte, Brazil. The study population consisted of individuals aged 60 years and over (older adults) who received at least one drug from PCU pharmacies between November 2013 and April 2014. The participants were selected non-randomly, and older adults were invited consecutively to participate in the study in PCU pharmacies. The exclusion criterion was the inability to communicate verbally or visually.

### Sample

The sample size was calculated from data in the computerized pharmacy system of the PCU. The mean monthly number of visits to these pharmacies by elderly people was 483. Based on the premise that dispensing of chronic care drugs from PCU pharmacies is done monthly, we considered that elderly people who were attended every month were part of the same population. Thus, by assuming that there was a finite population of 483 older adults, with a prevalence value of 50% for all observed characteristics, and taking a significance level of 5%, confidence interval of 95% and loss or refusal rate of 10%, the sample size was estimated to be 237 older adults. The “Open Epi” version 3.01 software was used for the sample size calculation.^14^

### Data collection and organization

Data were collected through face-to-face interviews, using a structured questionnaire that was applied by pharmacists and by pharmacy and medical students who had previously received training. The questions were on sociodemographic, clinical, functional, access-related and medicine-use characteristics. These data were complemented by consulting the medical records. The information on access to medicines covered the period of the last 30 days. The database was created using the Epi Info version 3.5.4 software (Centers for Disease Control and Prevention, Atlanta, United States). Quality control relating to data entry was performed by replicating 10% of the interviews. Reliability analysis among the data input typists was performed through kappa statistics, which showed a mean value of 1.0, thus indicating ideal agreement. These analyses were performed using the Statistical Package for the Social Sciences 25.0 software (SPSS 25.0).

### Variables

The dependent variable was full free access within the Brazilian National Health System to the drugs prescribed in the last 30 days. Free access included access to all prescription drugs. The category of free access included medicines received at PCUs and other services of the Municipal Health Department of Belo Horizonte, at specialized component pharmacies and through the *Saúde Não Tem Preço* (Health Doesn’t Have A Price) program at private pharmacies. Specialized component pharmacies are public utilities that provide medicines for specialized care. Under the *Saúde Não Tem Preço* program, users obtain drugs from private pharmacies without co-payment fees, with costs funded by the Ministry of Health.

The numbers of drugs obtained from public-system pharmacies and private pharmacies were ascertained established. The drugs included in the municipal essential medicines list (MEML) of Belo Horizonte were identified and then classified in accordance with level one of the World Health Organization’s Anatomical Therapeutic Chemical (ATC) system. The independent variables were divided as follows: (i) sociodemographic: gender, age, education, skin color, income and marital status (reclassified as with or without a partner); (ii) clinical characteristics: multimorbidity (≥ 2 diseases),^15^ classification of self-reported comorbidities, depressive symptoms and self-perceived health; (iii) functional characteristics: cognition, basic activities of daily living (BADL) and instrumental activities of daily living (IADL); and (iv) pharmacotherapeutic: polypharmacy (use of five or more prescription drugs).

Scales that had been validated or adapted for the Brazilian context were used to evaluate the following variables: depression - 15-item Geriatric Depression Scale (individuals with depressive symptoms: ≥ 6 points);^16^ cognition - Mini-Mental State Examination (individuals with cognitive disability were defined as those presenting ≤ 13 points if they were illiterate; ≤ 18 points if they had had one to eight years of schooling; and ≤ 24 points if they had had more than eight years of schooling);^17^^,^^18^ basic activities of daily living - Katz scale;^19^ and instrumental activities of daily living - Lawton and Brody scale (independent individuals = 21 points).^20^

Self-perceived health was measured by asking the patient: “In general, compared with other people of your age, would you say that your health is excellent, very good, good, fair or poor?” A positive assessment would comprise the responses “excellent”, “very good” or “good”, whereas a negative assessment would include the other answers, namely, “fair” or “poor”.

### Statistical analysis

Descriptive analysis was performed by determining the relative and absolute frequencies of categorical variables, and the median, interquartile range (IQR), minimum (min) and maximum (max) of quantitative variables. The association between free full access to prescription drugs within the Brazilian National Health System and the independent variables was analyzed using Pearson’s chi-square test. Continuous variables were dichotomized based on medians or definitions in the literature.

Variables with P ≤ 0.20 in univariate analyses were included in a multivariate logistic regression, and those with P ≤ 0.05 were retained in the final model. The goodness-of-fit of the final model was evaluated using the Hosmer-Lemeshow test (good fit if P > 0.05). Data analysis was performed using the SPSS 25.0 software.

### Ethical issues

The research project was approved by the research ethics committees of a public university and of the Municipal Health Department of Belo Horizonte on August 8, 2013, through protocol number CAAE 17339713.40000.5149. The older adults who agreed to participate signed an informed consent statement.

## RESULTS

The characteristics of the 227 elderly people who participated in the study are shown in Table 1. Their median age was 70 years (IQR = 12; minimum = 60 and maximum = 93); most were female (70.9%) and the majority had monthly income below two minimum wages (60.9%). These older adults predominantly presented preserved cognition (86.1%), independence in relation to BADL (76.5%) and partial independence in relation to IADL (77.1%). None of these elderly people were classified as totally dependent in relation to performing BADL or IADL.

**Table 1.** Description of older adults attended at the two healthcare centers (n = 227*)*

Variables	Free-access medicines		General description
	Yes	No	
	n (%)	n (%)	n (%)
Sociodemographic factors			
Gender			
Female	57 (35.4)	104 (64.6)	161 (70.9)
Male	34 (51.5)	32 (48.5)	66 (29.1)
Age			
≤ 70 years	53 (46.5)	61 (53.5)	114 (50.2)
> 70 years	38 (33.6)	75 (66.4)	113 (49.8)
Skin color			
Black	20 (45.5)	24 (54.5)	44 (19.5)
Other	70 (38.5)	112 (61.5)	182 (80.5)
Schooling			
> 4 years	41 (37.3)	69 (62.7)	110 (48.7)
≤ 4 years	49 (42.2)	67 (57.8)	116 (51.3)
Married			
Yes	42 (48.8)	44 (51.2)	86 (38.4)
No	48 (34.8)	90 (65.2)	138 (61.6)
Income			
> 2 minimum monthly wages	34 (40.5)	50 (59.5)	84 (39.1)
≤ 2 minimum monthly wages	51 (38.9)	80 (61.1)	131 (60.9)
Functional factors			
Cognition			
Preserved	79 (41.1)	113 (58.9)	192 (86.1)
Suspected disability	12 (38.7)	19 (61.3)	31 (13.9)
Instrumental activities of daily living			
Independent	25 (48.1)	27 (51.9)	52 (22.9)
Partially dependent	66 (40.1)	136 (59.9)	175 (77.1)
Basic activities of daily living			
Independent	73 (42.2)	100 (57.8)	173 (76.5)
Dependent ≥ one activity	18 (34.0)	35 (66.0)	53 (23.5)
Clinical factors			
Multimorbidity			
Yes	78 (37.3)	131 (62.7)	209 (92.1)
No	13 (72.2)	5 (27.8)	18 (7.9)
Arterial hypertension			
Yes	79 (40.3)	117 (59.7)	196 (86.3)
No	12 (38.7)	19 (61.3)	31 (13.7)
Diabetes mellitus			
Yes	24 (32.4)	50 (67.6)	74 (32.6)
No	67 (43.8)	86 (56.2)	153 (67.4)
Asthma and chronic obstructive pulmonary disease			
Yes	10 (29.4)	24 (70.6)	34 (15.0)
No	81 (42.0)	112 (58)	193 (85.0)
Depressive symptoms			
Yes	17 (34.0)	33 (66.0)	50 (22.5)
No	74 (43.0)	98 (57.0)	172 (77.5)
Self-perceived health			
Positive	63 (39.9)	95 (60.1)	158 (69.9)
Negative	28 (41.2)	40 (58.8)	68 (30.1)
Pharmacotherapeutic factors			
Polypharmacy			
Yes	44 (33.1)	89 (66.9)	133 (58.6)
No	47 (50.0)	47 (50.0)	94 (41.4)

Regarding clinical characteristics, the median number of self-reported diseases was three (IQR = 2), and 92.1% had multimorbidity. The most frequent diseases reported by these elderly people were hypertension (86.3%), musculoskeletal diseases (34.4%) and diabetes (32.6%). A positive self-perception of health was reported by 69.9% of the participants.

Regarding pharmacotherapeutic characteristics, the median number of medications used by these elderly people was five (IQR = 3; minimum = 1 and maximum = 13), and 58.6% were using polypharmacy.

**Figure 1.**
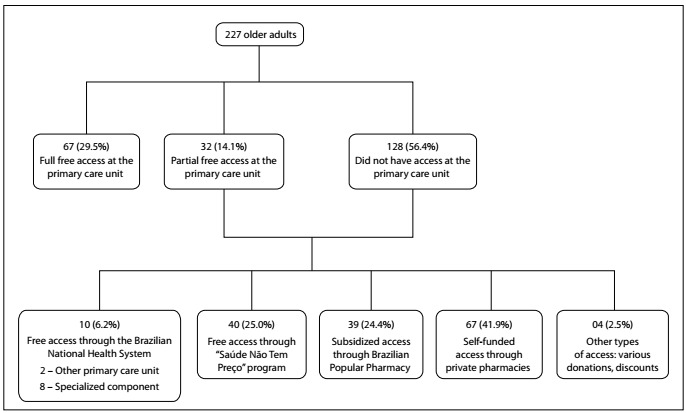
Characterization of access to medicines among the 227 older adults.

Figure 1 shows the characterization of access to medicines among these 227 older adults. Among them, 67 (29.5%) reported having full access to all medicines at the PCU pharmacies investigated. Access to at least one drug in the PCU pharmacies surveyed was reported by 32 (14.1%) of these older adults. We found that 128 (56.4%) of these elderly people had bought or received at least one drug through a channel other than the PCU.

The analysis on medicines that were not accessed at PCU pharmacies showed that the access strategies most used by the elderly people were the following: acquisition at a private pharmacy with the total amount paid by the user (41.9%); free access through the *Saúde Não Tem Preço* program (25.0%); and access through the popular pharmacy program, with acquisition subsidized by the Ministry of Health with co-payment fees (23.4%), as shown in Figure 1. Full access to the prescribed drugs was observed.

In total, 91 (40.1%) of these older adults had free full access to prescription drugs within the Brazilian National Health System. The median proportion of prescribed medications that were provided through the Brazilian National Health System was 83% (IQR = 33; minimum = 17 and maximum = 100). The median number of medicines accessed through the Brazilian National Health System was 4 (IQR = 3), and the median number of medicines accessed through private pharmacies was 1 (IQR = 1; minimum = 1 and maximum = 7). All the drugs used by 179 (78.9%) of these older adults were included in MEML.

The drugs used by the elderly people were predominantly from the following anatomical groups (level 1) of the ATC classification: group C - circulatory system (50.5%); group A - alimentary tract and metabolism (17.4%); and group N - nervous system (12.9%). On the other hand, analysis on drugs that were not included in RENAME and which were accessed through private pharmacies showed that the most frequent ATC groups were the following: group C (41.4%); group N (20.0%); and group B - blood and hematopoietic organs (12.9%) (Table 2). Univariate analysis on the associations between full access to medicines within the Brazilian National Health System and the independent variables (Table 3) showed that associations with the following factors were present at a 5% significance level: female, age ≤ 70 years, married, polypharmacy, multimorbidity and cardiovascular disease.

**Table 2.** Medicines acquired outside of the municipal essential medicines list (MEML)

Anatomical Therapeutic Chemical classification	All medicines		Medicines not included in the municipality essential medicines list	n	%
	n	%	Drug name		
Group A - Alimentary tract and nutrition	206	17.4		8	11.4
Drugs for peptic ulcer and gastroesophageal reflux disease			Pantoprazole	1	
Constipation drugs			Senna	1	
Insulin and analogues			Insulin glargine	2	
Blood glucose-lowering drugs, excluding insulin			Glimepiride	1	
			Sitagliptin	2	
Vitamins A and D, including associations			Vitamin D	1	
Group B - Drugs acting on blood and hematopoietic organs	95	8.0		9	12.9
Antithrombotic agents			Clopidogrel	4	
			Dabigatran	1	
			Cilostazol	1	
Vitamin B12 and folic acid			Cyanocobalamin	3	
Group C - Circulatory system drugs	597	50.5		29	41.4
Low-power thiazide diuretics			Chlorthalidone	2	
			Indapamide	3	
Hair-stabilizing agents			Diosmin Troxerutin	1 1	
Beta-blocking agents			Pindolol	1	
			Metoprolol Nebivolol	3 1	
Lipid-modifying agents			Atorvastatin Pravastatin Rosuvastatin	6 1 5	
			Fenofibrate	2	
Other cardiac preparations			Ivabradine	1	
Agents acting on the renin-angiotensin system			Telmisartan	2	
Group D - Dermatological drugs	8	0.7		2	2.9
Chemotherapy agents for topical use			Silver sulfadiazine	1	
Enzymes			Collagenase	1	
Group G - Genitourinary system and sex hormones	1	0.1	-	-	-
Group H - Systemic hormones, except sex hormones and insulins	43	3.6		2	2.9
Corticosteroids for systemic use			Fludrocortisone	1	
Anti-thyroid preparations			Thiamazole	1	
Group J- Anti-infective drugs for systemic use	14	1.2	-	-	-
Group M - Drugs acting on the musculoskeletal system and gout	43	3.6		4	5.7
Non-steroidal anti-inflammatory and anti-rheumatic products			Glucosamine	1	
			Meloxicam	1	
Central-action agents, muscle relaxants			Carisoprodol	1	
Drugs affecting bone structure and mineralization			Risedronate	1	
Group N - Nervous system drugs	153	12.9		14	20
Opioids			Codeine Tramadol	1 1	
Antiepileptics			Lamotrigine	1	
Antipsychotics			Pimozide	1	
Anxiolytics			Alprazolam	1	
Hypnotics and sedatives			Nitrazepam	1	
Antidepressants			Citalopram Escitalopram Sertraline	3 1 2	
Anti-vertigo preparations			Betahistine	1	
Group R - Respiratory system drugs	19	1.6	Nasal decongestants	1	1.4
Group S - Sensory system drugs	3	0.3	Artificial tears	1	1.4
Total	1,182	100		70	100

**Table 3.** Univariate and multivariate analysis on the factors associated with free full access to medicines within the Brazilian National Health System

Description	Full free access		Univariate analysis		Multivariate analysis	
Variable	Yes n (%)	No n (%)	Odds ratio (95% CI)	P-value	Odds ratio (95% CI)	P-value
Sociodemographic factors						
Gender						
Female	57 (35.4)	104 (64.6)	0.52 (0.289-0.922)	0.024		
Male	34 (51.5)	32 (48.5)	1			
Age						
≤ 70 years	53 (46.5)	61 (53.5)	1.72 (1.003-2.932)	0.048	1.92 (1.092-3.380)	0.024
> 70 years	38 (33.6)	75 (66.4)	1		1	
Income						
> 2 minimum monthly wages	34 (40.5)	50 (59.5)	1.07 (0.610-1.867)	0.821		
≤ 2 minimum monthly wages	51 (38.9)	80 (61.1)	1			
Living alone						
No	70 (40.5)	103 (59.5)	1.07 (0.571-1.997)	0.837		
Yes	21 (38.9)	33 (61.1)	1			
Married						
Yes	42 (48.8)	44 (51.2)	1.79 (0.323-0.968)	0.037		
No	48 (34.8)	90 (65.2)	1			
Functional factors						
Instrumental activities of daily living						
Independent	25 (48.1)	27 (51.9)	1.529 (0.819-2.854)	0.181		
Partially dependent	66 (40.1)	136 (59.9)	1			
Basic activities of daily living						
Independent	73 (42.2)	100 (57.8)	1.419 (0.746-2.702)	0.285		
Dependent in at least one activity	18 (34.0)	35 (66.0)	1			
Clinical factors						
Self-perceived health						
Positive	63 (39.9)	95 (60.1)	0.947 (0.531-1.689)	0.855		
Negative	28 (41.2)	40 (58.8)	1			
Arterial hypertension						
Yes	79 (40.3)	117 (59.7)	1.069 (0.492-2.325)	0.866		
No	12 (38.7)	19 (61.3)	1			
Diabetes mellitus						
Yes	24 (32.4)	50 (67.6)	0.616 (0.44-1.103)	0.102		
No	67 (43.8)	86 (56.2)	1			
Cardiovascular diseases						
Yes	24 (31.2)	53 (68.8)	0.561 (0.314-1.002)	0.049		
No	67 (44.7)	83(55.3)	1			
Asthma and chronic obstructive pulmonary disease						
Yes	10 (29.4)	24 (70.6)	0.576 (0.261-1.271)	0.168		
No	81 (42.0)	112 (58)	1			
Multimorbidity						
Yes	78 (37.3)	131 (62.7)	0.229 (0.079-0.667)	0.04	0.26(0.087-0.775)	0.016
No	13 (72.2)	5 (27.8)	1			
Pharmacotherapeutic factors						
Polypharmacy						
Yes	44 (33.1)	89 (66.9)	0.494 (0.287-0850)	0.01	0.47(0.268-0.838)	0.01
No	47 (50.0)	47 (50.0)	1		1	

Hosmer and Lemeshow test: chi-square = 4.780; degrees of freedom = 4; P = 0.311.

In the final logistic regression model, the presence of polypharmacy (odds ratio, OR = 0.47; confidence interval, CI = 0.268-0.838) and multimorbidity (OR = 0.26; CI = 0.087-0.775) was negatively associated with full access to medicines within the Brazilian National Health System. Age ≤ 70 years (OR = 1.92; CI = 1.092-3.380) was positively associated with full access to medicines in the Brazilian Unified Health System (Table 3).

## DISCUSSION

This study showed that 40% of the older adults surveyed had free full access to prescription drugs within the Brazilian National Health System. Given the greater burden of noncommunicable diseases among elderly people, ensuring access to drugs for this age group in order to provide more effective control of these diseases, thereby contributing towards improving the capacity of actions provided to this population to resolve problems, is a priority. Among the guidelines and constitutional principles of the Brazilian State is that it guarantees comprehensive therapeutic care. Therefore, access to medicines is a citizen’s right.^4^

The proportion of individuals with free full access to medicines within the Brazilian National Health System has ranged from 45.1% to 50.0% in investigations based on data obtained from the National Survey on Drug Access, Use and Promotion of Rational Use^4^ and the National Household Sample Survey (NHSS).^9^^,^^11^ The prevalence findings from the present study are in line those from other Brazilian studies, but comparisons should be made with caution because of methodological variations and inclusion criteria, given that the drug access survey and the study by Boing^11^ using NHSS data did not include older adults alone.

Despite the strategies implemented to conform with the guidelines of the National Drug Policy and the National Pharmaceutical Services Policy, investigations have shown that only about half of citizens with prescription drugs fully obtain them from the Brazilian National Health System.^4^ An economic evaluation from the perspective of the public healthcare system showed that medicines supplied through the primary public healthcare services of municipalities in the state of Minas Gerais had lower cost than those supplied through the *Aqui tem Farmácia Popular do Brasil* (The Brazilian popular pharmacy program is here) program, in which the Ministry of Health subsidizes the drug cost and the user pays co-payment fees for certain drugs purchased from private pharmacies.^21^ New evaluations on the effectiveness of expanding drug access policies such as free treatment for hypertension, diabetes and asthma through the popular pharmacy program may provide elements for increasing free access to medicines within the Brazilian National Health System.

Full access to medicines through healthcare centers was reported by around one-third of the older adults surveyed here. This shows that the availability of access needs to be streamlined to ensure drug provision and improve geographical accessibility. Thus, it is crucial to improve the management of pharmaceutical care to increase the effectiveness of free access to drugs for the population, and to improve the availability of medicines in public healthcare facilities.^4^^,^^7^ Improved management will contribute towards easing the challenge that the Brazilian National Health System faces in ensuring universal and continuous access to medicines, with equity and a capacity to resolve problems for the population

Most of the medicines used by the elderly people studied here were included in the municipal essential medicines list (MEML). This is a positive aspect of the way in which pharmaceutical services have been organized. It contributes towards implementation of access expansion actions, given that this facilitates the scheduling and dispensing steps. It also contributes towards rational use of drugs, since medicines included in the MEML are generally safe and effective.

A study conducted in primary healthcare centers in Belo Horizonte found a positive association between presence of the drug in the MEML and user access to it in the PCU.^22^ On the other hand, an analysis on drugs used by older adults that were not included in the MEML showed that from the perspective of elderly people’s care, therapeutic gaps existed. Absences of drugs such as cyanocobalamin, citalopram, clopidogrel, sertraline and escitalopram, which do not have substitutes in the drug list of the municipality investigated, were identified. In providing care for older adults, it is essential to develop actions to promote rational use of medicines and to implement strategies to ensure access to safe medications that are appropriate for the specificities of drug therapy in this population group. It is essential to consider the specificities of the older adult population^23^ in selecting drugs and also in relation to other stages of pharmaceutical services.

The feminization of aging,^24^ as well as the greater use of medicines by women, explain the lower likelihood of free access to medicines that was found in our study. On the other hand, the higher likelihood of free access among married older adults that was also observed illustrates the notion that family support can contribute towards healthcare. This increases the likelihood of access through more significant support for seeking drug provision strategies.

Cardiovascular diseases contribute significantly towards the burden of disease among older adults.^4^^,^^25^ They require the use of multiple medications, which thus reduces the likelihood of free full access. Some elderly people with cardiovascular diseases may require drugs that are not included in the MEML, in order to streamline therapeutic endpoints, and this also reduces the likelihood of access. Circulatory system drugs, along with those that act on blood and hematopoietic organs, are among the drugs excluded from the MEML, yet these drugs are widely used by older adults.

Free access to medications independently showed a direct association with age ≤ 70 years and indirect associations with polypharmacy and multimorbidity. The associations observed in relation to these factors can be explained by the fact that elderly people with higher numbers of chronic diseases demand more meaningful use of medicines, which reduces the likelihood of access to prescribed drugs.^13^ Understanding the specificities of drug therapy among older adults, the determinants of their drug use and the factors associated with access is vital for enabling development of actions towards greater availability of drugs and for improving the quality of pharmaceutical services for this population group within the Brazilian National Health System.

One strength of this study was that it analyzed the factors associated with free access to medicines through considering the pharmacotherapeutic, clinical and sociodemographic aspects of access. Thus, it provided elements to support evaluation of the way in which pharmaceutical services are organized within the Brazilian National Health System. This study therefore helps towards reducing the challenge of ensuring universal, equitable and problem-resolving access.

This study has some limitations. Firstly, it was conducted only in two PCUs in a single Brazilian city, which thus does not allow generalizations. Secondly, only older adults who attended the PCU to receive medications were selected for the study. This may have led to selection bias, with greater inclusion of individuals with lower degrees of frailty. Thirdly, the evaluation of access to medicines covered only the prescriptions held at the time of the interview. It may have been the case that, at that time, these older adults did not have all the prescriptions for the medicines that they were using, which would have induced bias regarding the number of medicines. Another limitation relates to the information about comorbidities, which was self-reported, thus reducing the quality of this clinical information.

Investigations on access to medicines are important because they provide elements for characterizing the healthcare system and for supporting policies and actions aimed at increasing access to priority groups such as older adults.^9^

## CONCLUSION

Free access to medicines is determined by the demographic, clinical, and pharmacotherapeutic characteristics of elderly people. Age ≤ 70 years increases the likelihood of free access, and older adults with multimorbidity and polypharmacy use have a lower likelihood of free full access to medicines. Access to medicines within the Brazilian National Health System among the elderly people surveyed here was high, but less than half of them were covered by full free access to prescription drugs. The availability of drugs in the PCUs was insufficient, which thus compromised the provision of drugs to older adults and geographical accessibility. Identification of factors associated with free access to medication among the elderly provides elements for guiding the Brazilian National Health System in implementing actions to improve access, such as the restructuring of pharmaceutical services to meet the specificities of the older adult population.
